# A Rare Case of Benign Long-Standing Ecchordosis Physaliphora

**DOI:** 10.7759/cureus.49490

**Published:** 2023-11-27

**Authors:** Yahea Alzahrani

**Affiliations:** 1 Department of Internal Medicine, Taif University, Taif, SAU

**Keywords:** ct, mri, chordoma, notochord, ecchordosis physaliphora

## Abstract

Ecchordosis physaliphora (EP) is a rare benign lesion arising from embryonic notochordal remnants, typically located in the retroclival region. This case report presents a 46-year-old male patient experiencing intermittent headaches and occipital pain. Imaging revealed a well-defined, smoothly corticated bony lesion on the left side of the clivus, accompanied by a characteristic bony stalk devoid of any aggressive features. A review of the patient's medical records indicated stable imaging findings of the lesion over six years. Clinicians and radiologists should be familiar with EP as a benign entity and differentiate it from aggressive pathologies.

## Introduction

Ecchordosis physaliphora (EP) is a rare congenital benign hamartomatous lesion originating from nodal cord remnants [1]. It is most commonly found in the retroclival prepontine region of the middle cranial fossa but can also be found anywhere along the midline from the skull base to the sacrum [2]. Ecchordosis physaliphora is typically asymptomatic but can occasionally cause mass effects with compression of the brain stem or cranial nerves [3].

Histopathologically, ecchordosis physaliphora is quite similar to chondroma [3]. The characteristic imaging findings of ecchordosis physaliphora include a well-demarcated, smoothly corticated bony defect in the clivus on computed tomography (CT) and a hypointense lesion on T1-weighted images and a hyperintense lesion on T2-weighted images, with no contrast enhancement on magnetic resonance imaging (MRI) [4].

A case of ecchordosis physaliphora is presented in this article with classic imaging characteristics, including the pathognomonic osseous stalk in both CT and MRI, with no change in size and appearance over a period of six years.

## Case presentation

**Figure 1:**
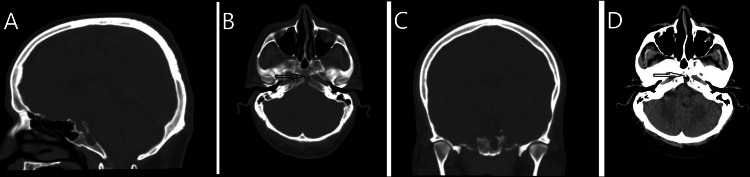
Non-enhanced brain CT Sagittal (A), axial (B), and coronal (C) CT scans of the brain on the bone window, along with an axial brain CT scan on the brain window (D), reveal a well-defined bony defect in the left side of the clivus. The margins of the defect are well-defined, smooth, and devoid of any signs of aggression. A subtle, tiny, stalklike hyperdense structure projecting from the clivus is also visible, which represents a pathognomonic osseous stalk (arrows on B and D). CT: computed tomography

A 46-year-old male patient presented with intermittent headache and occipital pain. The patient was afebrile with stable vital signs upon initial evaluation. Laboratory tests showed no abnormalities, and the neurological examination was unremarkable. A non-enhanced brain CT (Figure 1) demonstrated a clearly defined osseous defect on the left side of the clivus. The margin of this defect is well-defined, smooth, and devoid of aggressive characteristics. A tiny, hyperdense stalklike structure, representing a pathognomonic osseous stalk, is also visible projecting from the clivus.

**Figure 2:**
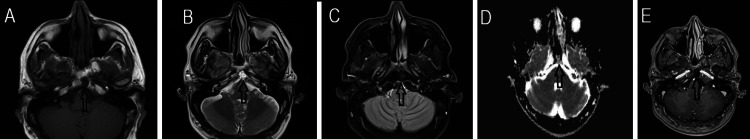
Brain MRI with and without contrast The study demonstrated a well-defined midline clival lesion (arrow) measuring 6 × 10 mm cystic lesion with hypointense signal on T1-weighted images (A) and hyperintense signal on T2-weighted images (B) and hypointense signal on FLAIR image (C). In addition, the lesion did not show restricted diffusion (D) and did not demonstrate abnormal contrast enhancement on T1-weighted post-contrast images (E). MRI: magnetic resonance imaging, FLAIR: fluid-attenuated inversion recovery

MRI (Figure 2) was performed with and without contrast. A well-defined midline cystic lesion measuring 6 × 10 mm was identified involving the dorsal aspect of the clivus. The lesion exhibited hypointensity on T1-weighted images, hyperintensity on T2-weighted images, and hypointensity following the cerebrospinal fluid (CSF) signal on fluid-attenuated inversion recovery (FLAIR) images. Diffusion-weighted imaging (DWI) revealed no evidence of restricted diffusion. Post-contrast T1-weighted images demonstrate no enhancement of the lesion. The remaining brain parenchyma appeared unremarkable.

On reviewing the patient's medical file, an older MRI and CT imaging examination six years earlier was available; it showed a stable size and appearance of this small midline clival lesion. Based on the imaging features and characteristics, the diagnosis of EP was established. Notably, the lesion remained stable in size and appearance over a period of six years, reinforcing the benign nature of EP.

## Discussion

Ecchordosis physaliphora (EP) is a benign lesion incidentally observed in skull base and spine imaging studies. It is a remnant of the notochord, a rod-shaped structure that is present during embryonic development and eventually forms the nucleus pulposus of the intervertebral discs [5]. EP is thought to occur in approximately 0.5%-2% of all autopsies and 1.5% of all brain MRIs but is often asymptomatic and goes unnoticed [1,6-8].

The diagnosis of EP is established based on characteristic imaging features of CT and MRI scans [3]. However, a CT scan is limited compared to an MRI in assessing EP for accurate localization and characteristics due to the small lesion size of EP and beam hardening artifacts in the posterior fossa images [1].

We presented a case of EP, which remains stable in size and appearance in imaging over a long period. The EP was incidentally discovered on a non-enhanced brain CT and enhanced brain MRI done in 2018, and the patient refused any further management and follow-up at that time.

On CT, EP typically appears as a well-demarcated, smoothly corticated bony defect in the clivus without aggressive features. Occasionally, a tiny, osseous, stalklike structure projecting from the clivus is considered a hallmark and pathognomonic feature of EP on CT scans [3]. EP is usually hypointense on T1-weighted magnetic resonance imaging (MRI) and hyperintense on T2-weighted MRI. It does not typically enhance with gadolinium contrast. One distinguishing feature of EP is the presence of a thin, T2-hypointense pedicle that attaches the mass to the underlying clivus. This pedicle is thought to represent the remnant of the notochordal canal [1,2,5-9].

According to Ilorah et al., atypical features of EP include an absent bony stalk, a T2-hypointense protrusion from the clivus, T2 hypointensities bordering the lesion, a T2-hypointense center within the lesion, and a T2 hyperintensity on the pharyngeal surface or dorsum sellae [10].

The most important differential diagnosis for EP is intradural chordoma, a rare malignant tumor that also arises from notochordal remnants. Chordomas are typically larger and more aggressive than EP, with imaging features including bone destruction, mass effect, and contrast enhancement [1,4]. Other differential diagnoses for EP include lipoma, meningioma, arachnoid cyst, Tarlov cyst, epidermoid cyst, dermoid cyst, and metastatic tumor [11].

Furthermore, radiologists and physicians should be aware of the anatomic variants of the skull base, including the craniopharyngeal canal, arrested sphenoid pneumatization, and asymmetric pneumatization petrous apex, as all do not need intervention or further imaging follow-up [12-14].

EP is typically a benign incidental finding and does not require treatment. However, in rare cases, EP may cause symptoms such as headache, neck pain, or cranial nerve palsies. These symptoms are usually caused by mass effects on the surrounding structures. If EP is causing symptoms, surgical resection may be necessary [15].

## Conclusions

The presented case of EP remained stable in size and appearance in imaging studies over an extended period. EP is a benign, incidental finding observed in imaging studies of the skull base and spine. It is crucial for radiologists and physicians to be familiar with the imaging characteristics of EP to accurately differentiate it from more aggressive lesions such as malignant chordoma. Follow-up examinations for EP patients would aid in establishing standardized diagnostic criteria and guidelines.
